# Discovery of Delirium Biomarkers through Minimally Invasive Serum Molecular Fingerprinting

**DOI:** 10.3390/metabo14060301

**Published:** 2024-05-26

**Authors:** Ana Viegas, Rúben Araújo, Luís Ramalhete, Cristiana Von Rekowski, Tiago A. H. Fonseca, Luís Bento, Cecília R. C. Calado

**Affiliations:** 1 ESTeSL—Escola Superior de Tecnologia da Saúde de Lisboa, Instituto Politécnico de Lisboa, Avenida D. João II, Lote 4.58.01, 1990-096 Lisbon, Portugal; ana.viegas@ulssjose.min-saude.pt; 2 Neurosciences Area, Clinical Neurophysiology Unit, ULSSJ—Unidade Local de Saúde São José, Rua José António Serrano, 1150-199 Lisbon, Portugal; 3 CHRC—Comprehensive Health Research Centre, Universidade NOVA de Lisboa, 1150-082 Lisbon, Portugal; rubenalexandredinisaraujo@gmail.com (R.A.);; 4 NOVA Medical School, Faculdade de Ciências Médicas, Universidade NOVA de Lisboa, 1169-056 Lisbon, Portugal; 5 ISEL—Instituto Superior de Engenharia de Lisboa, Instituto Politécnico de Lisboa, R. Conselheiro Emídio Navarro 1, 1959-007 Lisbon, Portugal; 6 Blood and Transplantation Center of Lisbon, Instituto Português do Sangue e da Transplantação, Alameda das Linhas de Torres, n° 117, 1769-001 Lisboa, Portugal; 7 iNOVA4Health—Advancing Precision Medicine, RG11: Reno-Vascular Diseases Group, NOVA Medical School, Faculdade de Ciências Médicas, Universidade NOVA de Lisboa, 1169-056 Lisbon, Portugal; 8 Intensive Care Department, ULSSJ—Unidade Local de Saúde São José, Rua José António Serrano, 1150-199 Lisbon, Portugal; 9 Integrated Pathophysiological Mechanisms, CHRC—Comprehensive Health Research Centre, NMS—NOVA Medical School, FCM—Faculdade de Ciências Médicas, Universidade NOVA de Lisboa, Campo Mártires da Pátria 130, 1169-056 Lisbon, Portugal; 10 iBB—Institute for Bioengineering and Biosciences, The Associate Laboratory Institute for Health and Bioeconomy (i4HB), Instituto Superior Técnico, Universidade de Lisboa, Av. Rovisco Pais, 1049-001 Lisbon, Portugal

**Keywords:** delirium, biomarkers, FTIR spectroscopy, serum, omics

## Abstract

Delirium presents a significant clinical challenge, primarily due to its profound impact on patient outcomes and the limitations of the current diagnostic methods, which are largely subjective. During the COVID-19 pandemic, this challenge was intensified as the frequency of delirium assessments decreased in Intensive Care Units (ICUs), even as the prevalence of delirium among critically ill patients increased. The present study evaluated how the serum molecular fingerprint, as acquired by Fourier-Transform InfraRed (FTIR) spectroscopy, can enable the development of predictive models for delirium. A preliminary univariate analysis of serum FTIR spectra indicated significantly different bands between 26 ICU patients with delirium and 26 patients without, all of whom were admitted with COVID-19. However, these bands resulted in a poorly performing Naïve-Bayes predictive model. Considering the use of a Fast-Correlation-Based Filter for feature selection, it was possible to define a new set of spectral bands with a wider coverage of molecular functional groups. These bands ensured an excellent Naïve-Bayes predictive model, with an AUC, a sensitivity, and a specificity all exceeding 0.92. These spectral bands, acquired through a minimally invasive analysis and obtained rapidly, economically, and in a high-throughput mode, therefore offer significant potential for managing delirium in critically ill patients.

## 1. Introduction

Despite the increase in the knowledge of biological mechanisms of diseases, delirium persists as an enduring challenge, particularly within Intensive Care Units (ICU), exerting substantial impacts on patient outcomes and placing immense strain on healthcare systems [1,2,3]. In the United States alone, delirium incurs a cost exceeding USD 38–152 billion annually [4]. Similarly, in a collective estimation across 18 European nations, it is predicted that the delirium burden surpasses USD 182 billion per year [5].

The vulnerability of critically ill patients to delirium is exacerbated by ICU interventions and environmental factors, such as prolonged invasive mechanical ventilation (IMV) and isolation [6,7,8,9]. This susceptibility was particularly accentuated during the coronavirus disease 2019 (COVID-19) pandemic, caused by severe acute respiratory syndrome due to coronavirus 2 (SARS-CoV-2), complicating patient care and extending recovery periods [10,11,12,13]. Indeed, epidemiological studies reveal a high variability in delirium prevalence [9], with notably higher rates of occurrence in ICU settings and, more recently, among patients with severe acute respiratory syndrome with SARS-CoV-2 infection, with rates reaching up to 80% in the most critically ill [14,15,16,17,18,19,20]. In resource-constrained ICU environments during the COVID-19 era, the frequency of delirium assessments often decreased due to the implementation of diagnostic and treatment strategies based on the available evidence. This reduction in assessments inadvertently contributed to an increase in delirium risk, severity levels, and duration, subsequently leading to heightened rates of adverse outcomes [10,14,17,21,22,23,24].

Classically defined as a disturbance in attention and awareness, delirium contrasts with baseline behavior, develops rapidly, and can fluctuate in severity throughout the day [25,26,27]. To date, it remains underdiagnosed and inadequately managed, partly due to this fluctuating nature, its varied presentation, the absence of a validated diagnostic tool for patients under IMV, and misconceptions regarding its inevitability in critically ill patients [13,28,29,30,31,32,33]. Moreover, traditional risk assessment tools for delirium, such as the Confusion Assessment Method for the ICU (CAM-ICU) [29], are limited by their reliance on subjective clinical evaluations and scoring systems, which may not always capture the fluctuating nature of delirium. The need for early discrimination between ICU patients with and without delirium is therefore critical to minimize morbidity and optimize healthcare resource allocation [24,34,35].

Hall et al. [36] reviewed the delirium biomarkers present in cerebrospinal fluid. Despite promising results, these analytes rely on an invasive method, making them unsuitable for routine analysis. Currently, non-invasive methods are being evaluated, such as those based on the analysis of cerebral perfusion acquired through near-infrared spectroscopy and/or transcranial Doppler. Although these methods show promise, they are not also applicable to routine analysis [37]. Therefore, identifying delirium biomarkers that can be obtained by minimally invasive procedures and analyzed by rapid and simple modes could enhance our understanding of the pathophysiological processes and significantly improve disease management [38].

Despite the well-established association of serum molecules like interleukin-6 (IL-6) with delirium, the translation of these biomarkers into robust predictive models has encountered obstacles, primarily due to delirium’s inherently complex and multi-dimensional characteristics [5,39,40,41]. For example, IL-6 presents odds ratio in the delirium population that range between 0.84 and 5.2 [38], while the C-reactive protein shows an odds ratio spanning from 0.45 to 5.73 [38], indicating the limited specificity of these biomarkers. 

New approaches based on omics sciences can pave the way for the identification of a delirium biomarker [39,41,42], as these sciences enable a holistic search among a whole set of biomolecules within a defined system. For example, Han et al. [39], through lipidomic and metabolomics analyses of cerebrospinal fluid, identified phosphatidylethanolamine (PE 40:7e) as critical, leading to a predictive model of delirium with an Area Under the Curve (AUC) of 0.92 on the Receiver Operating Characteristic curve. However, the models were developed using data from only ten patients with delirium, all of whom were elderly with hip fractures, representing a homogeneous clinical phenotype. Moreover, the analysis was performed on cerebrospinal fluid, which was obtained through a highly invasive procedure. Huang et al. [43] performed a metabolomics analysis of serum, which is collected using a minimally invasive method. Their study resulted in a prediction model based on three molecules that forecast delirium, with an AUC of 0.72, despite the inclusion of a relatively homogeneous cohort consisting of 58 elderly patients undergoing cardiac surgery. In addition to the limitations mentioned above, these techniques are complex and time-consuming, which may limit their application to large-scale studies and, consequently, their translation into clinical practice [44]. 

Fourier-Transform InfraRed (FTIR) spectroscopy presents a range of characteristics that can help overcome the aforementioned limitations. As a vibrational spectroscopic method, it captures the molecular fingerprint of the system, which can be associated with a specific metabolic status with high sensitivity and specificity [45,46,47,48,49]. Furthermore, the analysis can be conducted using less than a drop of blood and can be carried out in high-throughput mode utilizing a micro-plate, such as a 96-well micro-plate. The analytical process also requires minimal sample processing, needing only a simple dehydration step, with each sample’s spectrum being acquired in less than two minutes [45,46].

The present work aims to evaluate how serum molecular fingerprints, obtained through FTIR spectroscopy, can be used to predict delirium in critically ill patients, based on the data of a cohort of 134 critically ill COVID-19 patients admitted to an ICU, of which 26 presented with delirium. The analysis was conducted in high-throughput mode using a 96-well micro-plate, following a simple serum dilution and dehydration. Therefore, this analysis relies on a biofluid obtained through a minimally invasive procedure, and is established based on a method that is rapid, economical, and capable of high-throughput processing, making it suitable for future large-scale population studies.

## 2. Materials and Methods

### 2.1. Population

A total of 134 patients admitted at the ICU of Hospital São José (Lisbon, Portugal) were considered, of which 26 were diagnosed with delirium, while 108 were not. All patients presented with a COVID-19 diagnosis, as determined via real-time polymerase chain reaction assay targeting SARS-CoV-2. None of these patients were classified as terminally ill or presented with a delirious state upon or prior to the ICU admission. Additionally, they had no history of major psychiatric disorders, and had not been administered benzodiazepines such as oxazepam and midazolam prior to their admission.

Delirium was assessed using the CAM-ICU [29]. The CAM-ICU score was determined by assessing the presence of four specific criteria to evaluate the patient’s cognitive state: acute onset or fluctuating course (1), inattention (2), disorganized thinking (3) and altered level of consciousness (4). Patients with a Richmond Agitation and Sedation Scale (RASS) score of −4 or −5 were considered ineligible for CAM-ICU screening while those exhibiting at least one positive CAM-ICU score during their ICU stay were diagnosed with delirium [29,50]. In cases where the delirium assessment was not performed, the patient’s delirium status was established based on a comprehensive review of clinical notes. This examination considered the administration of neuroleptics, the RASS score and the Diagnostic and Statistical Manual of the American Psychiatric Association (DSM-5) criteria [25].

This study received approval from the Hospital Ethics Committee, Unidade Local de Saúde São José (ULSSJ), and informed consent was secured from each patient or their family members for data collection before participation. The patients’ clinical information was accessed through the hospital’s electronic medical records. Clinical data were anonymized.

### 2.2. Demographics and Other Clinical Characteristics

Patient demographics, such as gender, age and body mass index (BMI), were examined (Table 1). Clinical variables assessed included comorbidities like arterial hypertension and diabetes mellitus. Considerations also encompassed the administration of midazolam and oxazepam, the use of IMV and Extracorporeal Membrane Oxygenation (ECMO), as well as the length of ICU stay and mortality rates in both the hospital and ICU settings (Table 1). Most patients (*n* = 127, i.e., 95%) were admitted to the ICU primarily due to acute respiratory distress syndrome or acute respiratory failure (Supplementary Tables S1 and S2). Other, less common, reasons for admission included sepsis (*n* = 1), septic shock (*n* = 1), convulsions (*n* = 1) or for monitoring purposes (*n* = 3).

### 2.3. Collection of Biological Samples

Peripheral blood was collected in a tube with no anticoagulant VACUETTE^®^, using standard blood collection procedures. To ensure consistency, all blood collections were performed during the first morning shift in the ICU, between 7 and 9 a.m. Once collected, the blood was immediately put on cold storage at −4 °C and processed within 2 h. Serum was then obtained by centrifugation at 3500 rpm for 10 min (Mikro 220T, Hettich, Tuttlingen, Germany) and maintained at −80 °C until further analysis.

### 2.4. FTIR Spectroscopy

Triplicates of 25 μL of serum, pre-diluted at 1/10 in water, from each sample, were pipetted to a 96-well Si plate and subsequently dehydrated for about 3.5 h in a desiccator under vacuum (Vacuubrand, ME 2, Wertheim, Germany). Spectral data were collected using an FTIR spectrometer (Vertex 70, Bruker) equipped with an HTS-XT (Bruker, Billerica, MA, USA) accessory. Each spectrum represented 64 coadded scans, with a 2 cm^−1^ resolution, and was collected in transmission mode, between 400 and 4000 cm^−1^. The first well of the 96-well plate did not contain a sample and the corresponding spectra were acquired and used as the background, according to the HTS-XT manufacturer.

### 2.5. Spectra Pre-Processing and Processing 

Spectra with atmospheric correction were subsequently submitted to a combination of the following pre-processing methods: baseline correction, unit-vector normalization, and a second derivative using a Savitzky–Golay filter, with a second-order polynomial over a 15-point window. The impact of spectra pre-processing was evaluated on a t-distributed, stochastic-neighbor-embedding (*t*-SNE) technique to develop Naïve-Bayes predictive models. Naïve-Bayes models were developed with and without implementing a Fast-Correlation-Based Filter (FBCF) for feature selection (i.e., spectral bands). A cross-validation method with 10 folds (90% training, 10% test size) was applied. 

Atmospheric compensation was conducted with OPUS^®^ software, version 6.5 (Bruker, Germany), whereas the remaining pre-processing and processing methods were conducted with Orange: Data Mining Toolbox [51], version 3.36.2 (Bioinformatics Lab, University of Ljubljana, Ljubljana, Slovenia). 

### 2.6. Other Statistical Analysis

For continuous variables, the independent samples t-test was employed to compare two groups when the data followed a normal distribution. In cases where the data were non-normally distributed, the Mann–Whitney U test was used as a non-parametric alternative. For target variables with multiple categories and normally distributed data, ANOVA was used. For the analysis of categorical data, chi-square tests were employed to assess independence within contingency tables. If the contingency table included cells with low expected frequencies, potentially compromising the validity of the chi-square results, Fisher’s exact test was utilized. Statistical significance was set for two-sided *p*-values of less than 0.05. 

Descriptive and inferential statistics were conducted with IBM SPSS Statistics software, version 27 (IBM Corp., New York, NY, USA).

## 3. Results and Discussion

### 3.1. General Characterization of the Population

Out of the 134 patients considered, 26 were diagnosed with delirium and 108 with non-delirium (Table 1). In this cohort, the prevalence of delirium was significantly higher among males (*p* = 0.029) and factors such as the use of IMV, a longer length of ICU stay and the administration of oxazepam and midazolam were associated with an increased risk of its development (*p* < 0.001). These findings align with other studies regarding the impact of gender and treatment on the occurrence and severity of delirium [3,6,10,34,52]. 

To develop robust prediction models for delirium, based on the serum molecular fingerprint, as captured by FTIR spectroscopy, a subgroup of 26 non-delirium patients was defined, ensuring there were no significant statistical differences in demographics and clinical variables between the two groups, with the exception of delirium status and oxazepam administration (*p*-value = 0.034) (Table 2).

### 3.2. Serum Spectral Analysis

The serum FTIR spectra were evaluated by univariate and multivariate data analyses.

#### 3.2.1. Univariate Spectral Analysis

**Figure 1:**
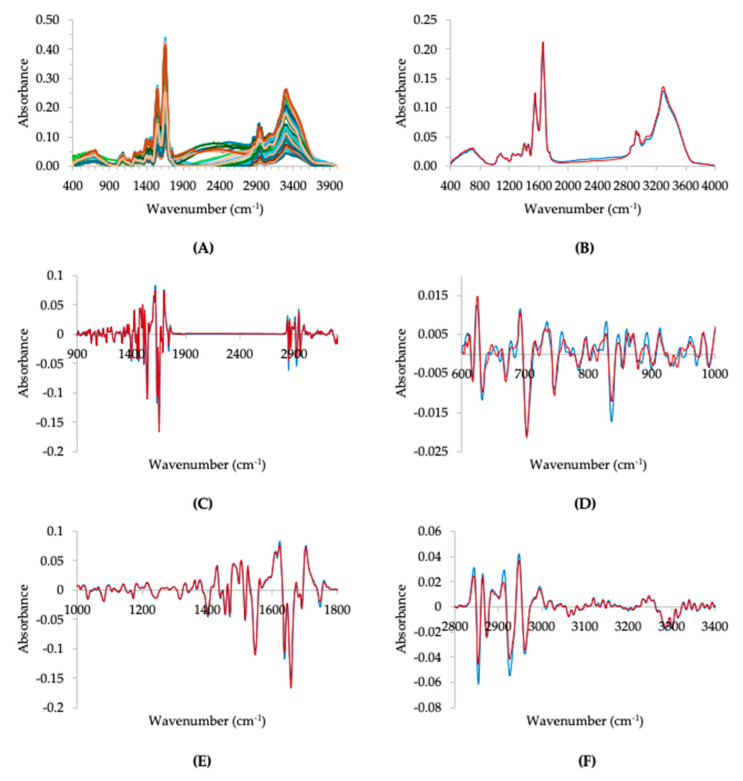
Serum spectra from delirium (*n* = 26) and non-delirium (*n* = 26) patients after baseline correction (**A**). Average serum spectra of delirium (blue line) and non-delirium patients (red line) after baseline correction and atmospheric compensation (**B**) or the average normalized second-derivative serum spectra (**C**–**F**).

Figure 1 represents both the non-derivate and derivative FTIR spectra of serum from delirium and non-delirium patients. The average FTIR spectra of 26 serum samples from delirium patients was very similar to the average spectra of 26 serum samples from non-delirium patients (Figure 1B). The differences between the average spectra of these two populations were highlighted by the normalized second-derivative spectra (Figure 1C–F), as expected, since the second derivative resolves overlapping spectral bands and normalization minimizes the impact of different light path lengths during measurements, thus underlining the differences in biochemical composition among the samples.

**Figure 2:**
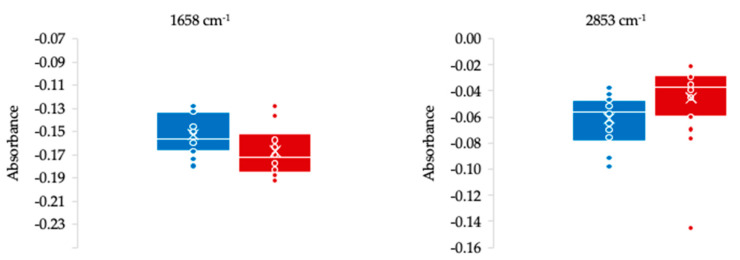
Boxplots of 1658 and 2853 cm^−1^ bands from the normalized second-derivative spectra of delirium (blue) and non-delirium patients (red).

Based on the average normalized second-derivative spectra of serum from delirium patients, a total of 41 negative bands were identified (Figure 1, Supplementary Table S3). A Student’s *t*-test was conducted to compare these bands between delirium and non-delirium patient groups, uncovering five statistically different bands (Table 3, Figure 2). Among these, bands at 2853, 2926 and 2961 cm^−1^ are associated with lipids, while the band at 1470 cm^−1^ is associated with both lipids and proteins. This association highlights the disruptive role of lipids in similar neurodegenerative diseases [43,53,54,55,56,57,58]. For instance, Huang et al. [43] observed significant changes in the lipid profiles of patients with post-operative delirium, hypothesizing that lipid dysregulation might contribute to the pathogenesis of the condition. Similarly, Guo et al. [59] found that deficiencies in ω3 and ω6 fatty acids and disruptions in the energy metabolism may diminish the brain’s inherent protective capacity, potentially contributing to the development of post-operative delirium. Furthermore, Han et al. [39] identified 33 lipids that were dysregulated in the pre-operative cerebrospinal fluid of patients who later developed post-operative delirium. The corresponding pathway enrichment analysis also highlighted significant perturbations in lipid metabolism pathways, including the glycerophospholipid metabolism and sphingolipid metabolism. All these data support previous hypotheses that lipids are essential for cell signaling and brain homeostasis, impacting neuronal membrane properties and signaling pathways [60,61]. Such changes in lipid metabolism can influence neuron function, potentially leading to acute neuropsychiatric disorders conditions like delirium by affecting fatty acid synthesis, membrane integrity, neuroinflammation, and neuron apoptosis [55,61,62,63,64,65,66,67,68,69,70,71]. 

Another relevant band, statistically different between the two groups, was the 1658 cm^−1^ associated with the amide I from proteins (Figure 2). This observation is consistent with the changes observed in various proteins in delirium patients [73], and the inflammatory process linked to the pathophysiology of delirium [74,75]. These inflammatory processes may indicate potential blood–brain barrier damage, which could affect neurotransmitters and brain functionality [76,77,78,79]. For example, Ritter et al. [75] observed alterations in serum protein levels, including elevated levels of cytokines (viz. STNFR1, STNFR2, adiponectin and IL-1β), among critically ill patients with delirium. These changes in protein levels signify an inflammatory response that could precipitate or exacerbate delirium, underlining the interplay between inflammation and acute cognitive dysfunction. Also, Han et al. [80], based on proteomic analysis, identified 63 dysregulated proteins in the cerebrospinal fluid of post-operative delirium patients, which are associated with several nervous system-related pathways. Various observations underscore the importance of neuroinflammatory processes in the development of delirium, emphasizing the crucial connection between protein dynamics and the mechanisms of the disease [37,81,82,83]. Indeed, a recent systematic review highlighted the significance of neuroinflammatory processes in delirium, identifying 370 peri-operative protein biomarkers implicated in immune system activation, inflammatory responses, and the coagulation cascade [73]. 

A Naïve-Bayes predictive model was developed based on the two spectral bands exhibiting the most significant differences between the two populations, specifically the 1658 and 2853 cm^−1^ bands (Figure 2). Nonetheless, the model presented very low specificity with an AUC < 0.65, indicating a low predictive performance.

#### 3.2.2. Multivariate Spectral Analysis

**Figure 3:**
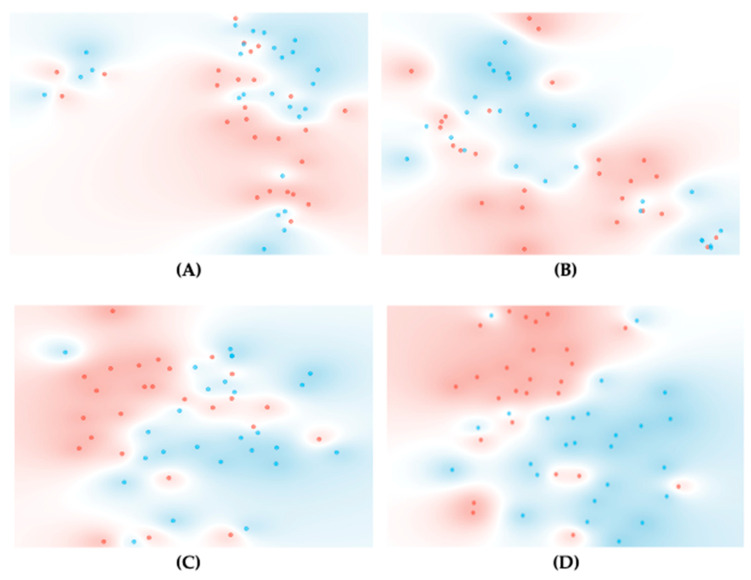
*t*-SNE for serum spectra of delirium (blue) and non-delirium patients (red) after the following spectra pre-processing methods: baseline correction (**A**); second derivate (**B**); normalized second derivative (**C**); normalized second derivative coupled with an FCBF, selecting 2912, 1149, 942, 1546, 1268, 987 and 1041 cm^−1^ (**D**).

A *t*-SNE was conducted to evaluate the impact of spectra pre-processing methods, including baseline correction and second derivative, with and without normalization (Figure 3). For the second derivative, certain spectral regions were disregarded to reduce noise amplification due to derivatives. The *t*-SNE serves as a valuable tool for visualizing high-dimensional data, as it condenses them into a lower-dimensional space while maintaining the intrinsic structure of the data. This characteristic makes *t*-SNE particularly adept at revealing clusters or groupings within intricate datasets. Interestingly, the *t*-SNE score plot clustered some scores according to patient groups (i.e., with and without delirium), with some improvements observed in the second-derivative spectra, probably due to bands’ resolution (Figure 3). Nonetheless, none of the pre-processing methods resulted in a clear class separation.

Subsequently, models predicting delirium were developed, employing the Naïve Bayes probability classification algorithm. These models are based on the entire spectral range or, in the case of using the second derivative, on specific regions spanning between 800–1800 and 2800–3400 cm^−1^ (Table 4). However, none of the evaluated pre-processing methods, including baseline correction or second derivative with and without normalization, yielded satisfactory models (AUC < 0.7). This is consistent with the *t*-SNE observations.

To enhance the model’s performance, FCBF was applied for feature selection (i.e., spectral bands), resulting in a notable improvement in the model’s performance for all the spectra pre-processing methods (Table 4). Of these models, the best ones were based on seven bands from the second-derivative spectra (2912, 1149, 942, 1546, 1268, 987 and 1041 cm^−1^), presenting with an AUC of 0.99 and a sensitivity and a specificity of 0.92 (Supplementary Figure S1). This result is consistent with the *t*-SNE analysis conducted on these seven bands, indicating a significantly improved cluster of scores between delirium and non-delirium patients compared to prior analyses focusing on a different spectral region (Figure 3D). This underscores the efficacy of *t*-SNE as a valuable preliminary tool in evaluating the impact of spectra pre-processing methods and spectral regions. 

The predictive model, utilizing seven FTIR spectral bands of serum, shows a superior performance compared to previous studies. For instance, Huang et al. [43], based on serum metabolomics, developed a predictive model for delirium with an AUC of only 0.72. The predictive model by Han et al. [39] showed an apparently better performance (AUC = 0.92); however, this model relied on cerebrospinal fluid obtained through a highly invasive procedure and was established on only 10 elderly patients with hip fracture, i.e., with a very homogenous population. In contrast, the present model was developed using serum, i.e., from a biofluid acquired through a minimally invasive procedure, unlike the cerebrospinal fluid. Additionally, it was based on a more heterogeneous population, specifically considering a range of ages (57 ± 15 years).

The best prediction model comprised seven bands, ranked in descending order of importance: 2912, 1149, 942, 1546, 1268, 987 and 1041 cm^−1^. The bands at 2912 and 1149 cm^−1^ are associated with lipids and carbohydrates, respectively, aligning with the known link between delirium and disruptions in the lipid metabolism and the energetic metabolism [39,40,41,43,59]. Indeed, these metabolic alterations serve as recognized indicators for post-operative delirium [39,43]. The bands at 942 and 1546 cm^−1^, associated with proteins, and the band at 1041 cm^−1^, linked to phosphate esters, along with those at 1268 and 987 cm^−1^, related to phosphate groups (which can also be attributed to phosphorylated proteins and lipids), can be associated with the neuroinflammation, metabolic imbalances and oxidative stress in delirium [20,84]. These observations corroborate the importance of the disruptions in lipid and energetic metabolism, as well as inflammatory processes, in the development of delirium. Furthermore, the identification of seven serum spectral bands using the best predictive model aligns with the complex nature of delirium, suggesting that a set of molecules likely serves as delirium biomarkers, as previously noted by e.g., Wiredu et al. [73], instead of one unique molecule.

## 4. Conclusions

In this study, predictive models for delirium were successfully developed with a heterogenous ICU population, identifying seven serum FTIR spectral bands that contributed to a predictive model with excellent performance metrics (AUC, sensitivity and specificity all exceeding 0.92). These spectral bands represent a wide range of molecules, including proteins, lipids and carbohydrates, thereby reflecting the complex and intricate mechanisms that underlie the development of delirium. This research underscores the importance of defining a set of biomolecules—as exemplified by the molecular fingerprint of serum captured through FTIR spectroscopy—rather than relying solely on individual biomarkers. Moreover, the outlined method offers the advantage of acquiring biomarkers through a minimally invasive technique, using blood rather than cerebrospinal fluid, and involves simple sample processing that only requires serum dilution with water and dehydration prior to spectra acquisition. Additionally, the spectra are acquired using a rapid, economical and high-throughput mode, making it suitable for future large-scale population studies. Ultimately, this study holds significant potential in identifying robust biomarkers for delirium using minimally invasive techniques, thereby facilitating rapid analysis and significantly enhancing the clinical management of delirium patients.

## Figures and Tables

**Table 1:** Demographic and clinical characteristics of 134 patients, of which 26 presented with delirium, and the *p*-value of the statistical analysis comparing the two groups.

	Delirium Patients (*n* = 26)	Non-Delirium Patients (*n* = 108)	*p*-Value	Statistic Test Used
Age (years) (mean)	55.38 ± 14.82	57.00 ± 16.46	0.469	Student’s *t*-test
Male gender (n)	22	67	0.029	Fisher’s exact test
BMI (mean)	27.42 ± 4.60	31.16 ± 23.88	0.408	Mann–Whitney U
Presence of comorbidities (n)	23	86	0.299	Fisher’s exact test
Arterial Hypertension (n)	12	46	0.427	Chi-square
Diabetes Mellitus (n)	7	26	0.449	Chi-square
Hospital death (n)	6	25	0.994	Chi-square
ICU death (n)	4	24	0.441	Fisher’s exact test
N. of days of ICU stay (mean)	25.42 ± 19.70	8.56 ± 9.25	<0.001	Mann–Whitney U
Use of IMV (n)	26	58	<0.001	Fisher’s exact test
Use of ECMO (n)	5	9	0.103	Chi-square
Oxazepam (n)	12	5	<0.001	Fisher’s exact test
Midazolam (n)	24	37	<0.001	Fisher’s exact test

**Table 2:** Demographic and clinical characteristics of 52 patients, of which 26 presented with delirium, and the *p*-value of the statistical analysis comparing the two groups.

	Delirium Patients (*n* = 26)	Non-Delirium Patients (*n* = 26)	*p*-Value	Statistic Test Used
Age (years) (mean)	55.38 ± 14.82	57.65 ± 15.87	0.806	Student’s *t*-test
Male gender (n)	22	20	0.726	Fisher’s exact test
BMI (mean)	27.42 ± 4.59	29.76 ± 6.44	0.189	Mann–Whitney U
Presence of comorbidities (n)	23	24	1.000	Fisher’s exact test
Arterial Hypertension (n)	14	12	0.782	Chi-square
Diabetes Mellitus (n)	8	8	1.000	Chi-square
Hospital death (n)	6	9	0.540	Chi-square
ICU death (n)	4	9	0.199	Fisher’s exact test
N. of days of ICU stay (mean)	25.46 ± 19.70	19.73 ± 13.00	0.081	Mann–Whitney U
Use of IMV (n)	26	25	1.000	Fisher’s exact test
Use of ECMO (n)	5	5	1.000	Chi-square
Oxazepam (n)	12	4	0.034	Fisher’s exact test
Midazolam (n)	24	23	1.000	Fisher’s exact test

**Table 3:** Average values and standard deviations for statistically significant spectral bands of the normalized second-derivative spectra of serum from delirium patients compared to non-delirium patients, along with their molecular assignments according to [72]. The corresponding *p*-values from Student’s t-test comparisons between the two populations are also presented.

	Delirium		Non-Delirium				
Bands (cm^−1^)	Average	Standard Deviation	Average	Standard Deviation	*p*-Value	Vibrational Mode	Functional Group/Biocompound
1470	−0.046	0.008	−0.041	0.008	0.046	δas(CH_3_) δas(CH_3_)	Lipid, protein
1658	−0.152	0.017	−0.167	0.022	0.012	80% ν(CO), 20% ν(CN)	Amide I peptide, protein
2853	−0.061	0.020	−0.045	0.028	0.025	νs(CH_2_)	Lipids
2926	−0.055	0.020	−0.041	0.022	0.027	νas(CH_2_)	Lipids
2961	−0.037	0.005	−0.034	0.004	0.043	νas(CH_3_)	Lipids

ν, stretching; δ, bending; as, antisymmetric; s, symmetric.

**Table 4:** Cross-validation performance of Naïve-Bayes prediction models for delirium, based on various spectral pre-processing techniques, based on the serum’s whole spectra, or between 800–1800 and 2800–3400 cm^−1^, or after bands’ selection by an FCBF.

Spectra Pre-Processing	Regions (cm^−1^)	AUC	Sensitivity	Specificity	Accuracy	Precision
Baseline correction	400–4000	0.678	0.538	0.538	0.538	0.538
Normalized and baseline		0.639	0.462	0.615	0.538	0.545
Second derivative	800–1800 and 2800–3400	0.583	0.423	0.462	0.442	0.440
Normalized second derivative		0.589	0.731	0.577	0.654	0.633
Baseline correction	438, 922 and 418	0.856	0.692	0.769	0.731	0.750
Normalized and baseline	841 and 3962	0.767	0.692	0.731	0.712	0.720
Second derivative	2868, 1587, 1149, 855, 1061, 1630 and 1792	0.900	0.846	0.885	0.865	0.880
Normalized second derivative	2912, 1149, 942, 1546, 1268, 987 and 1041	0.989	0.923	0.923	0.923	0.923

## Data Availability

The datasets used and/or analyzed during the current study are available from the corresponding author on reasonable request. The data are not publicly available due to their use in an ongoing research project.

## Supplementary Materials

The following supporting information can be downloaded at: https://www.mdpi.com/article/10.3390/metabo14060301/s1, Table S1: Reasons for ICU admission of the 134 patients, of which 26 presented with delirium, and the *p*-value from the statistical analysis comparing these two groups based on a Fisher’s exact test. Table S2: Reasons for ICU admission of the 52 patients, of which 26 presented with delirium, and the *p*-value from the statistical analysis comparing the two groups, based on a Fisher’s exact test. Table S3: Bands without statistically significant differences between patients with delirium and non-delirium, as indicated by the respective *p*-values. Figure S1: ROC curve of the cross-validation of Naïve-Bayes prediction models based on seven spectral bands selected by an FCBF, of normalized second-derivative spectra.
