# Supplementary material for: Discovery of Delirium Biomarkers through Minimally Invasive Serum Molecular Fingerprinting
Source: Metabolites. 2024 May 26;14(6):301. doi: 10.3390/metabo14060301 (PMC11205956; doi:10.3390/metabo14060301)
Supplement: Supplementary file 1 [file metabolites-14-00301-s001.zip › Supplementary files.pdf]

Table S1: Reasons for ICU admission of the 134 patients, of which 26 presented with delirium, and the *p*-value from the statistical analysis comparing these two groups based on a Fisher's exact test.

ICU admission reason	**Delirium patients (n=26)**	**Non-delirium patients (n=108)**	***p*-value**
Acute respiratory distress syndrome; Acute lung injury (n)	1	2	0.479
Acute Respiratory failure (n)	1	2	0.479
Acute respiratory failure due to COVID-19 (n)	23	98	0.724
Convulsions (n)	1	0	0.194
For monitoring (n)	0	3	1.000
Non-hemorrhagic hypovolemic shock (n)	0	1	1.000
Sepsis (n)	0	1	1.000
Septic shock (n)	0	1	1.000

Table S2: Reasons for ICU admission of the 52 patients, of which 26 presented with delirium, and the *p*-value from the statistical analysis comparing the two groups, based on a Fisher's exact test.

ICU admission reason	**Delirium patients (n=26)**	**Non-delirium patients (n=26)**	***p*-value**
Acute respiratory distress syndrome; Acute lung injury (n)	1	2	1.000
Acute Respiratory failure (n)	1	0	0.491
Acute respiratory failure due to COVID-19 (n)	23	24	1.000
Convulsions (n)	1	0	0.491
For monitoring (n)	0	1	1.000
Non-hemorrhagic hypovolemic shock (n)	0	0	-
Sepsis (n)	0	0	-
Septic shock (n)	0	0	-

Table S3: Bands without statistically significant differences between patients with delirium and non-delirium, as indicated by the respective *p*-values.

Spectral regions (cm^{-1})	Bands (cm^{-1})	*p*-value
900 to 1000	930	0.639
1001 to 1800	1033	0.604
	1082	0.515
	1174	0975
	1244	0.495
	1317	0.812
	1403	0.148
	1442	0.182
	1456	0.736
	1470	0.046
	1518	0.587
	1548	0.074
	1638	0.118
	1685	0.943
	1691	0.864
	1748	0.097
2800 to 3400	2853	0.025
	2873	0.670
	2961	0.043
	3009	0.110
	3030	0.250
	3062	0.711
	3073	0.579
	3211	0.477
	3266	0.287
	3287	0.161
	3301	0.656
	3313	0.656
	3332	0.466
	3346	0.111
	3358	0.465

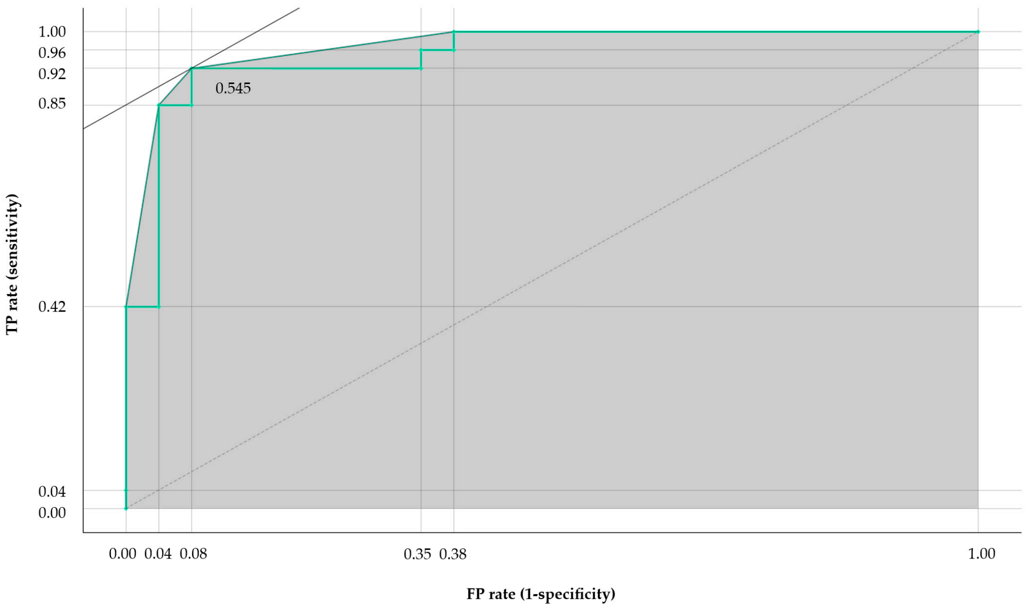

Figure S1: ROC curve of the cross-validation of Naïve-Bayes prediction models based on seven spectral bands selected by an FCBF, of normalized second-derivative spectra.
