# Supplementary figures and images for: Discovery of Delirium Biomarkers through Minimally Invasive Serum Molecular Fingerprinting
Source: Metabolites. 2024 May 26;14(6):301. doi: 10.3390/metabo14060301 (PMC11205956; doi:10.3390/metabo14060301)

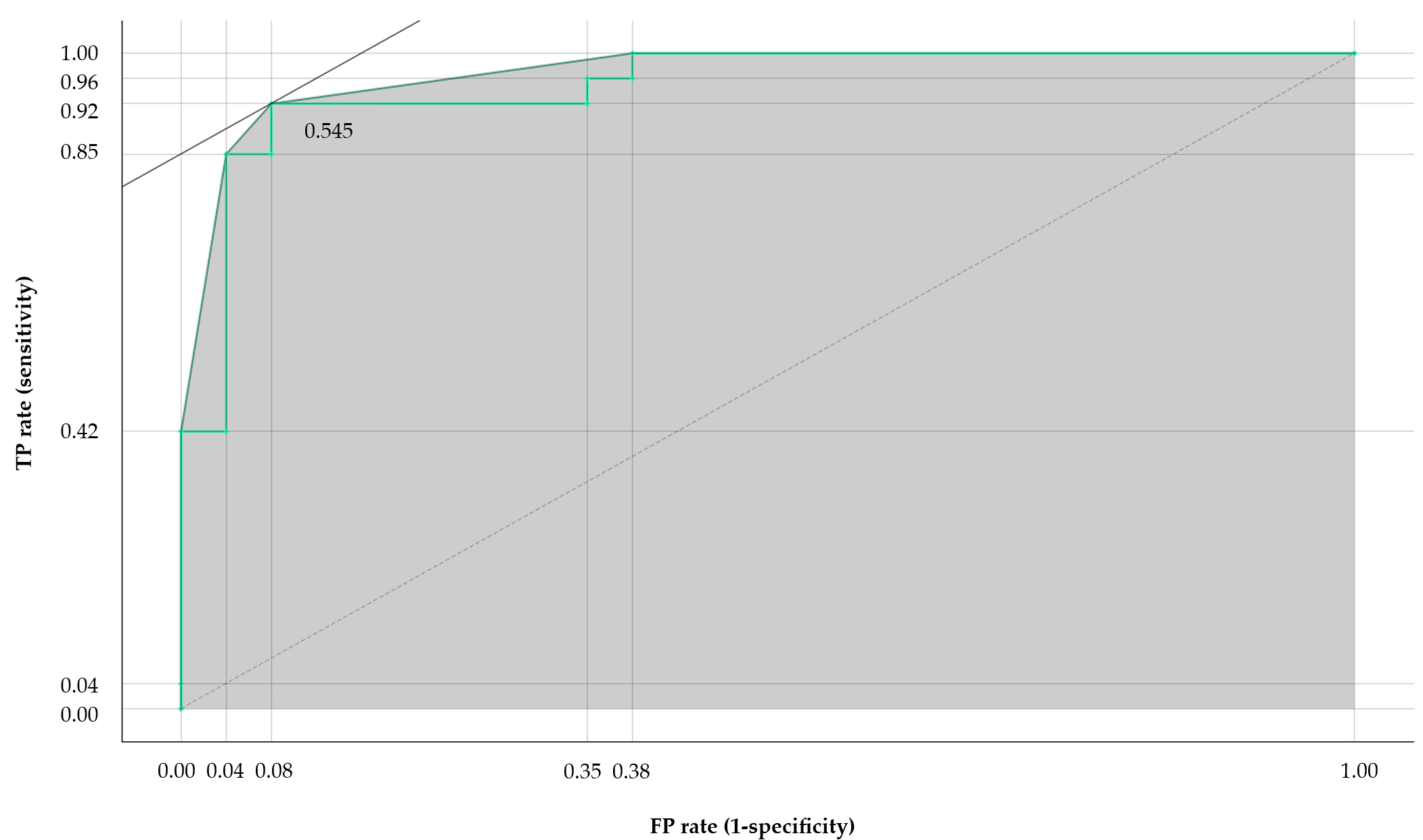

Supplement: Supplementary file 1 [file metabolites-14-00301-s001.zip › Supplementary Figure S1.jpg]

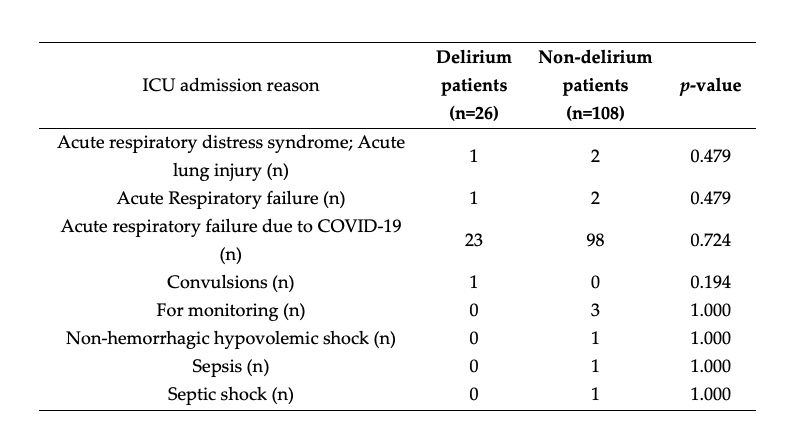

Supplement: Supplementary file 1 [file metabolites-14-00301-s001.zip › Supplementary Table S1.jpg]

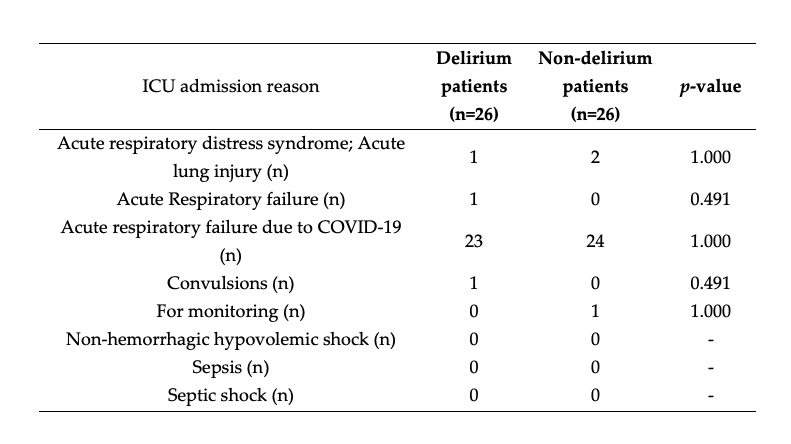

Supplement: Supplementary file 1 [file metabolites-14-00301-s001.zip › Supplementary Table S2.jpg]

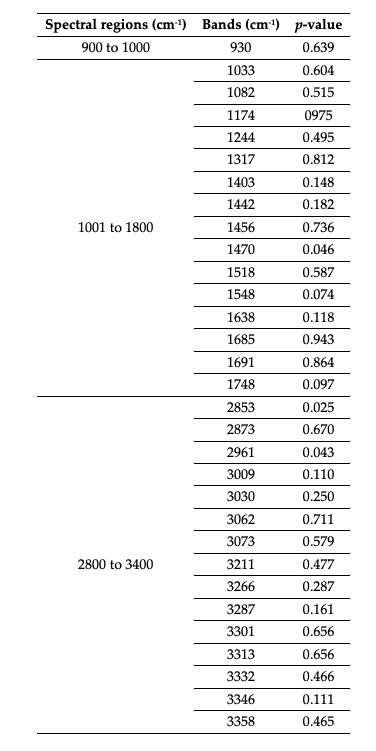

Supplement: Supplementary file 1 [file metabolites-14-00301-s001.zip › Supplementary Table S3.jpg]
